# Work loss and activity impairment due to extended nausea and vomiting in patients with breast cancer receiving CINV prophylaxis

**DOI:** 10.1007/s00520-023-08119-1

**Published:** 2023-10-25

**Authors:** Lee Schwartzberg, Rudolph M. Navari, Kathryn J. Ruddy, Thomas W. LeBlanc, Rebecca Clark-Snow, Rita Wickham, Dwight Kloth, Gary Binder, William L. Bailey, Marco Turini, Ravi Potluri, Xing Liu, Eros Papademetriou, Eric J. Roeland

**Affiliations:** 1 Renown Institute for Cancer, Reno, NV USA; 2 World Health Organization, Atlanta, GA USA; 3 Mayo Clinic, Rochester, MN USA; 4 Duke University School of Medicine, Duke Cancer Institute, Durham, NC USA; 5 Oncology Supportive Care Consultant, Overland Park, KS USA; 6 Rush University College of Nursing, Chicago, IL USA; 7 Fox Chase Cancer Center, Philadelphia, PA USA; 8 Servier Pharmaceuticals, Boston, MA USA; 9 Helsinn Therapeutics (U.S.), Inc., Iselin, NJ USA; 10 Helsinn Healthcare SA, Lugano, Switzerland; 11 Putnam Associates, New York, NY USA; 12 Oregon Health and Sciences Center, Knight Cancer Institute, Portland, OR USA

**Keywords:** Work loss, Breast cancer, CINV, Chemotherapy, Antiemetic

## Abstract

**Purpose:**

Chemotherapy-induced nausea and vomiting (CINV)’s impact on work loss remains poorly described. We evaluated associations between the duration of CINV episodes, CINV-related work loss (CINV-WL), and CINV-related activity impairment (CINV-AI) in patients with breast cancer receiving highly emetogenic chemotherapy.

**Methods:**

We analyzed data from a prospective CINV prophylaxis trial of netupitant/palonestron and dexamethasone for patients receiving an anthracycline and cyclophosphamide (AC) for breast cancer (NCT0340371). Over the observed CINV duration (0–5 days), we analyzed patient-reported CINV-WL and CINV-AI for the first two chemotherapy cycles. We categorized patients as having either extended (≥ 3 days) or short (1–2 days) CINV duration and quantified its impact on work using the Work Productivity and Activity Impairment Questionnaire (WPAI).

**Results:**

Overall, we captured data for 792 cycles in 402 women, including 136 (33.8%) employed patients with 35.3% reporting CINV. Of those with CINV, patients reported CINV-WL in 26 cycles and CINV-AI in 142 cycles. Of those with CINV, 55.3% of extended CINV cycles experienced CINV-WL compared to 16.7% of short CINV cycles (*p* < 0.001). The relative risk of CINV-WL between extended and short CINV was 3.32 (*p* < 0.01) for employed patients. The mean difference in CINV-AI scores (higher = worse) between extended and short duration CINV was 5.0 vs. 3.0 (*p* < 0.001).

**Conclusion:**

Extended (≥ 3 days) CINV was associated with more than triple the risk of CINV-WL and higher CINV-AI compared with short CINV.

**Supplementary Information:**

The online version contains supplementary material available at 10.1007/s00520-023-08119-1.

## Introduction

While evidence describes chemotherapy-induced nausea and vomiting (CINV)’s influence on quality of life, including impairment of daily activities [1–3], its impact on work loss for patients with cancer is less clear. Studies measuring work loss primarily use it as a proxy for indirect cost, with varying levels of impact noted [4–6]. Generally, CINV’s impact on daily activities determines patients’ decision to continue treatment [7–9]. Therefore, CINV’s impact on work loss and activity impairment is essential to understand but remains poorly described.

The common primary endpoint in CINV clinical trials, complete response, assesses vomiting or the use of rescue medications during a 5-day period starting at chemotherapy administration. However, as a binary variable, it does not address CINV’s duration. Extended duration CINV (≥ 3 days) may cause more clinical consequences than shorter CINV, particularly given evidence suggesting a correlation between duration and severity [10], and the impact of extended duration CINV on quality of life [11] and recurrent CINV [12]. Given this difference in severity, quality of life, and recurrence, CINV duration may also have variable impacts on work loss and activity impairment. Consequently, as pre-specified measures within a clinical trial, we sought to evaluate the relationship between CINV duration and its relation to work loss and activity impairment using a validated patient-reported outcome.

## Methods

As a pre-specified secondary analysis, we assessed patient-reported CINV up to 5 days after chemotherapy administration from a prospective, multi-cycle CINV prophylaxis trial of oral or intravenous netupitant/palonestron (NEPA) and dexamethasone (12mg day 1 only) for patients with breast cancer receiving anthracycline with cyclophosphamide (AC) (NCT0340371). The trial’s primary results have been previously reported [13], demonstrating both intravenous and oral NEPA are effective and safe. We combined the available data from both arms of the study for this secondary analysis.

We defined CINV using complete response, including vomiting (expulsion of stomach contents through the mouth) or retching (an attempt to vomit that is not productive of stomach contents), or the use of a rescue medication (any medication given to alleviate emetic symptoms, except for contraindicated NK1-receptor antagonists and 5-HT3 receptor antagonists). Complete response was captured using daily patient diaries from 0 to 120 h (5 days) after the start of chemotherapy for each cycle. Patients recorded the following: (1) the date and time of each emetic episode (retching or vomiting), (2) the date and time of rescue medication intake, (3) the name of the rescue medication taken, and (4) the daily nausea intensity assessed by a visual analogue scale. The study team checked the completed diaries on day 6 of each cycle. Patients without an emetic episode or rescue medication use were defined as achieving a complete response for that chemotherapy cycle.

We categorized CINV duration as extended (≥ 3 out of 5 days) or short (1–2 out of 5 days) regardless of whether the days were consecutive based on prior reports [10, 11]. Here we report on the first 2 cycles, in which work loss and activity impairment data were collected for all patients with active employment. Pre-specified endpoints included patient-reported CINV-associated work loss using the Work Productivity and Activity Impairment: Specific Health Problem survey [14, 15], and CINV-related impaired activity [0 (none)—10 (worst) Likert scale] for chemotherapy cycles 1 and 2 per the daily activity question from the WPAI:SHP survey. Of note, the WPAI:SHP specifically asks patients to consider work and productivity loss associated with their CINV and identifies hourly levels of overall work loss for all causes; the former aspect of the tool was used in this analysis (Supplement 1). CINV-related work loss and activity impairment could involve nausea with or without vomiting or rescue medication use. Both are rated from 0 to 10 with a maximum score of 10 indicating worse impact. To mirror the National Cancer Institute’s Patient-Reported Outcomes version of the Common Terminology Criteria for Adverse Events (PRO-CTCAE) classifying CINV by severity [16], which used a score of 4 on a 5-point scale to indicate “severe”, we used and a cutoff of ≥ 8 on the 10-point scale of the WPAI to indicate severe CINV-related activity impairment.

### Statistical analysis

We analyzed patient demographics, CINV, and related work loss and activity impairment using descriptive statistics (t-tests for work loss and Mann–Whitney U for activity impairment as it is an ordinal outcome). Relative risk (RR), chi-square statistics, and t-tests were used for significance testing of rates between courses with extended (≥ 3 days) CINV vs. short (1–2 days) CINV. Analyses were conducted by cycle and in aggregate. All study analyses were conducted using SAS software 9.4 and Microsoft Excel.

## Results

The trial’s CONSORT diagram has been reported previously [13]. We captured data on CINV-related work loss and activity impairment for 792 first and second cycles in 402 women, including 136 (33.8%) employed patients. Employed patients provided data for 255 cycles with CINV-related work loss and activity impairment data. The overall mean age was 55.4 years (SD = 9.8). Employed patients were younger than non-employed patients (employed mean age = 51.0 years, SD = 8.0; non-employed mean age = 57.6 years, SD = 9.9, *p* < 0.0001). Over 97% of patients completed two cycles of chemotherapy, including for those with and without CINV and employed status (Table 1). CINV was observed in 173 (21.8%) of these 792 cycles (i.e., the complete response rate of these cycles was 78.2%). Overall, 92 (53.2%) of cycles with CINV had a short duration and 81 (46.8%) had extended duration.

**Table 1:** Group demographics

	Overall	NV Occurrence^a^			NV Occurrence, among employed patients		
		No NV	NV	*P*-value	No NV	NV	*P*-value
	*N* = 402	*N* = 281	*N* = 121		*N* = 88	*N* = 48	
Age (years)							
Mean (SD)	55.4 (9.8)	56.5 (9.7)	52.8 (9.6)	0.0005	51.7 (8.1)	49.8 (8.1)	0.1797
Median (Q1—Q3)	56.0 (49.0–62.0)	57.0 (50.0–64.0)	52.0 (47.0–60.0)	0.0003	51.5 (46.5–57.5)	50.5 (45.0–54.5)	0.2405
Min, Max	27.0, 81.0	27.0, 80.0	28.0, 81.0		34.0, 80.0	29.0, 69.0	
Age group, *n*(%)				0.0369			0.9489
18—49 years	107 (26.6%)	67 (23.8%)	40 (33.1%)		36 (40.9%)	21 (43.8%)	
50—64 years	227 (56.5%)	159 (56.6%)	68 (56.2%)		48 (54.5%)	25 (52.1%)	
65 + years	68 (16.9%)	55 (19.6%)	13 (10.7%)		4 (4.5%)	2 (4.2%)	
Sex, *n* (%)							
Female	402 (100.0%)	281 (100.0%)	121 (100.0%)		88 (100.0%)	48 (100.0%)	
Country, *n* (%)				< 0.0001			< 0.0001
USA	82 (20.4%)	28 (10.0%)	54 (44.6%)		9 (10.2%)	22 (45.8%)	
Georgia	60 (14.9%)	45 (16.0%)	15 (12.4%)		6 (6.8%)	6 (12.5%)	
Ukraine	181 (45.0%)	150 (53.4%)	31 (25.6%)		51 (58.0%)	11 (22.9%)	
Russia	79 (19.7%)	58 (20.6%)	21 (17.4%)		22 (25.0%)	9 (18.8%)	
Race, *n* (%)				0.0169			0.5177
White	375 (93.3%)	269 (95.7%)	106 (87.6%)		83 (94.3%)	43 (89.6%)	
Black or African American	13 (3.2%)	6 (2.1%)	7 (5.8%)		3 (3.4%)	4 (8.3%)	
American Indian or Alaska Native	1 (0.2%)	1 (0.4%)	0 (0.0%)		–	–	
Asian	2 (0.5%)	1 (0.4%)	1 (0.8%)		–	–	
Native Hawaiian or Other Pacific Islander	1 (0.2%)	1 (0.4%)	0 (0.0%)		–	–	
Other	1 (0.2%)	1 (0.4%)	0 (0.0%)		1 (1.1%)	0 (0.0%)	
Unknown	9 (2.2%)	2 (0.7%)	7 (5.8%)		1 (1.1%)	1 (2.1%)	
Number of Cycles, *n* (%)				0.8042			0.9427
1 only	12 (3.0%)	8 (2.8%)	4 (3.3%)		2 (2.3%)	1 (2.1%)	
2	390 (97.0%)	273 (97.2%)	117 (96.7%)		86 (97.7%)	47 (97.9%)	
Any work loss overall, *n* (%)	96 (70.6%)	–	–		63 (71.6%)	33 (68.8%)	0.7282
Hours of work loss overall							
Mean (SD)	29.8 (16.1)	–	–		31.3 (13.8)	27.0 (19.7)	0.2197
Median (Q1—Q3)	32.0 (20.0–40.0)	–	–		35.0 (24.0–40.0)	24.0 (12.0–36.0)	0.0661
Min, Max	1.0, 104.0	–	–		1.0, 72.0	3.0, 104.0	
Any work loss due to CINV, *n* (%)	33 (24.3%)	–	–		16 (18.2%)	17 (35.4%)	0.0251
Hours of work loss due to CINV							
Mean (SD)	20.5 (14.0)	–	–		22.4 (15.2)	18.8 (13.0)	0.4593
Median (Q1—Q3)	20.0 (8.0–32.0)	–	–		19.0 (9.0–40.0)	22.0 (6.0–28.0)	0.4477
Min, Max	1.0, 48.0	–	–		1.0, 40.0	3.0, 48.0	
Any work loss for other reasons, *n* (%)	75 (55.1%)	–	–		51 (58.0%)	24 (50.0%)	0.3727
Hours of other work loss							
Mean (SD)	29.1 (14.5)	–	–		31.6 (12.0)	23.8 (17.9)	0.0297
Median (Q1—Q3)	32.0 (20.0–40.0)	–	–		32.0 (24.0–40.0)	20.0 (10.0–37.0)	0.0162
Min, Max	4.0, 80.0	–	–		4.0, 72.0	4.0, 80.0	

a) Occurrence of NV was identified based on either the use of anti-emetic rescue medication or retching/vomiting occurring within 5 days of the start of the cycle 1 or 2

### CINV-related work loss

**Fig. 1:**
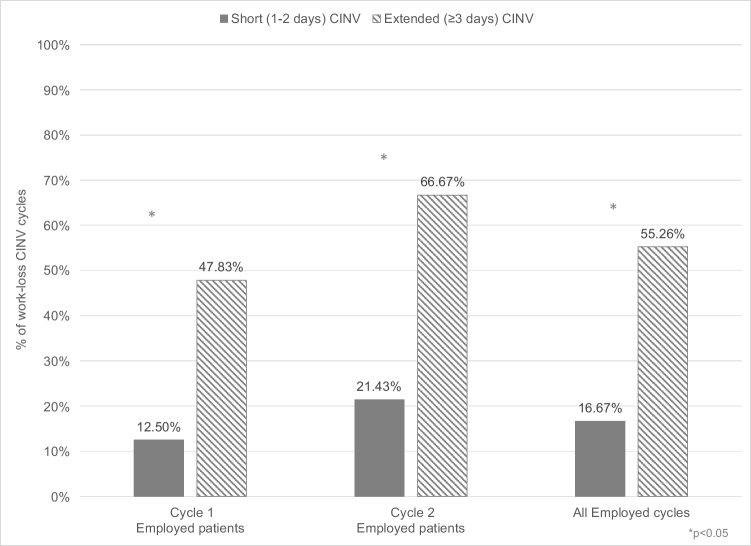
Percent of CINV-related work loss by CINV duration and cycle among employed patients with breast cancer receiving AC over two cycles

Patients reported CINV-related work loss in 26 cycles; specifically, in 3.3% of all cycles, 15.0% of cycles with CINV, and 38.2% of employed patient cycles with CINV. Results were also similar at the patient level (4.2% of all patients, 14.1% of patients with any CINV, 35.4% of employed patients with any CINV). These patients experienced an average (SD) of 20.5 (13.6) hours of work loss and a median (range) of 20 (8–32) hours per cycle. When we categorized cycles by CINV duration, CINV-related work loss in cycle 1 was observed in 47.8% (11/23) of employed patients with extended CINV compared to 12.5% (2/16) with short CINV (RR 3.83, p = 0.03; Fig. 1). CINV-related work loss in cycle 2 was seen in 66.7% (10/15) of employed patients with extended CINV and 21.4% (3/14) with short CINV (RR 3.11, *p* = 0.01; Fig. 1). Across both cycles, CINV-related work loss was observed in 55.3% (21/38) of cycles with extended CINV vs. 16.7% (5/30) for short CINV (RR 3.32, *p* = 0.001; Fig. 1). However, the mean work hours lost was not different between patients with extended (19.8, SD = 12.6) or short CINV (23.4, SD = 18.8; *p* = 0.606).

### CINV-related activity impairment

**Fig. 2:**
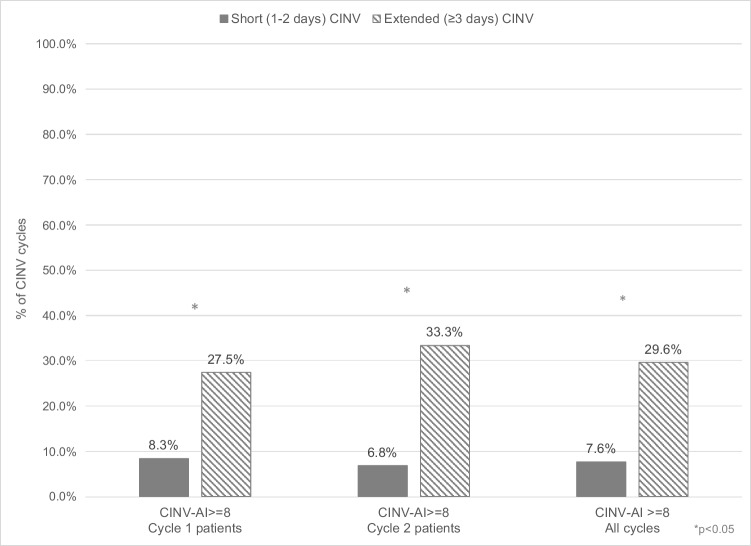
Percent of CINV-related activity impairment by CINV duration and cycle among employed patients with breast cancer receiving AC over two cycles

A total of 142 cycles (17.9% of all, 82.1% of cycles with CINV) and 103 patients (25.6% of all, 85.1% of patients with CINV) involved CINV-related activity impairment. The mean CINV-related impairment score was 4.7 (2.9) (out of a maximum score of 10), and the median score (range) was 4 (2–7). The median CINV-related impaired activity scores was 5.0 for extended CINV and 3.0 for short CINV (*p* < 0.01). Patients with extended CINV were more likely to have more severe CINV-related activity impairment, defined as an impairment score ≥ 8/10. In cycle 1, 27.5% (14/51) of patients with extended CINV had severe impact on activity impairment compared to 8.3% (4/48) with short CINV (RR: 3.33; *p* = 0.01; Fig. 2). In cycle 2, 33.3% (10/30) of patients with extended CINV had a severe score of activity impairment compared to 6.8% (3/44) of patients with short CINV (RR: 4.89; *p* = 0.003; Fig. 2). Across both cycles, 29.63% (24/81) of patients with extended CINV had a severe activity impairment score compared to 7.61% (7/92) of short CINV (RR: 3.89; *p* = 0.0002; Fig. 2).

## Discussion

We found that extended (≥ 3 days) compared to short (1–2 days) CINV duration was associated with more than triple the risk of CINV-related work loss and nearly four times the risk of CINV-related activity impairment. We used prospectively collected data using a validated patient-reported outcome from the first two cycles of a CINV prophylaxis trial in women with breast cancer receiving AC. Specifically, we demonstrated that working women experienced CINV-related work loss in 55.3% of cycles with extended CINV compared to 16.7% for short CINV (RR: 3.32; *p* = 0.001). We also found that 29.6% (24/81) of working women with extended CINV experienced severe activity impairment compared to 7.6% with short CINV (RR: 3.89; *p* = 0.0002). Overall, Patients with extended CINV had markedly higher rates of CINV-related work loss and impaired activity compared to CINV that only lasted 1–2 days – a finding consistent within each cycle and in aggregate. This duration-dependent response suggests that extended CINV deserves additional attention as a causative factor for work loss and activity impairment associated with chemotherapy. Duration of CINV may be an important measure of patient outcomes beyond the current regulatory defined assessment period. Collectively, this adds to the growing evidence that CINV duration has variable impacts on work loss and activity impairment in addition to CINV’s impact on nausea and vomiting-related severity [10], quality of life [11], and risk of recurrence [12].

Despite evidence-based guidelines demonstrating decreased CINV-related symptoms and impact [8, 17–19], patients with cancer consistently report CINV as among the most concerning cancer treatment-related symptoms. Data demonstrate that acute care related to CINV is avoidable [20–22]. Mindful of CINV’s impact on avoidable acute care, The Centers for Medicare and Medicaid Services has included it within Medicare’s OP-35 oncology outcome measure [23], now publicly reported and used to increase or reduce hospitals’ Medicare reimbursement. This inclusion recognizes the potential to prevent CINV and its related outcomes. The traditional complete response measure is commonly evaluated in the acute, delayed, or overall period, which does not distinguish between patients with short and extended duration of CINV. Duration and severity are common and well-accepted measures of chemotherapy symptoms and are included within the US National Cancer Institute’s PRO-CTCAE measures of chemotherapy toxicities [24]. Ballatori reported, “At a unifactorial analysis, both intensity and duration of CINV were found significant in determining the impact of CINV on patients’ daily lives, but at a multifactorial analysis adjusting for the effect of each variable on the other, only duration remained significant. This finding suggests that the duration of CINV should be assessed with an accuracy much greater than that currently used.” [25] Roeland et al. also identified an association between nausea duration and severity, with patients experiencing short (1–2 days) of CINV duration patients reporting lower nausea severity scores than those with extended (≥ 3 days) CINV duration, suggesting that duration may be a surrogate measure for severity [10].

We found CINV-related work loss in over one-third of employed patients with CINV and related impaired activity in over 80% of all patients with CINV. Prior studies assessing quality of life that have included work loss as a component have not been consistent. In a prospective German study, Ihbe-Heffinger et al. reported three patients losing an average of 4.4 full workdays due to CINV but noted that this finding “may not be readily applicable to other healthcare settings.” [4] In a US-based study, Tina Shih et al. reported from an exploratory observational study using insurance claims and productivity data that, on average, patients experiencing CINV lost 6.23 full workdays per month (the number of patients with work loss was not reported) [6]. The first study indicates a rather small effect of CINV on work loss, whereas the second study indicates a larger effect. Recognizing the challenges of working during the first two cycles of AC, the current study showed that 17 of 33 employed patients with CINV lost a mean of 20.5 h over 5 days. A higher proportion of patients were affected by work loss, but the number of hours lost was lower than in the other studies. This finding may be confounded by the shorter observation period (5 vs. 30 days) over two cycles of chemotherapy. The finding that CINV affected work loss in women participating in a clinical trial is notable as these patients are more likely given more comprehensive care and support than patients in the real world; yet, more than one-third experienced work loss. Given the negative effects CINV has on cancer treatment adherence [7, 9], the work loss implications may also factor into patients’ treatment decisions.

Strengths of this analysis include the use of a large controlled prospective study with pre-specified evaluations of the days of CINV as well as validated, patient-reported measures of work loss and activity impairment explicitly associated with CINV. Additionally, we focused on patients with the highest risk of CINV, including women with breast cancer receiving AC. Limitations include the lack of a comparative arm with another Neurokinin-1 receptor antagonist to confirm that the impact of CINV duration is a class effect, and the limited number of patients who were employed who could be evaluated for work loss. Furthermore, the evaluation of CINV-related work loss for the 5-day period after chemotherapy over two cycles may understate the impact of any lost work occurring beyond that time. Finally, while CINV-related work loss is addressed explicitly in the Work Productivity and Activity Impairment: Specific Health Problem patient-reported survey, a patient may have difficulty discerning how much of their work loss was caused by CINV versus other chemotherapy-related side effects.

## Conclusions

Even with guideline-concordant CINV prophylaxis in all patients over two cycles of chemotherapy in this prospective study, CINV occurred in 22% of AC cycles. The majority of CINV lasted 1–2 days. Yet, extended (≥ 3 days) CINV duration was associated with more than triple the risk of work loss and over 80% greater risk of severe activity impairment compared with short CINV. For employed patients, nearly 40% of CINV led to work loss during days over the 5 days of a chemotherapy cycle. CINV-related activity impairment occurred in 82% of all patients with CINV. These data suggest that CINV duration may be a meaningful measure of its clinical and economic impact in addition to its impact on severity, quality of life, and risk of recurrence.

### Supplementary Information

Below is the link to the electronic supplementary material.

Supplementary file1 (DOCX 124 KB)

## Data Availability

Requests for the clinical trial data can be directed to datasharing@helsinn.com.
