# Supplementary figures and images for: Work loss and activity impairment due to extended nausea and vomiting in patients with breast cancer receiving CINV prophylaxis
Source: Support Care Cancer. 2023 Oct 25;31(11):654. doi: 10.1007/s00520-023-08119-1 (PMC10600031; doi:10.1007/s00520-023-08119-1)

Supplement 1. WPAI Questionnaire


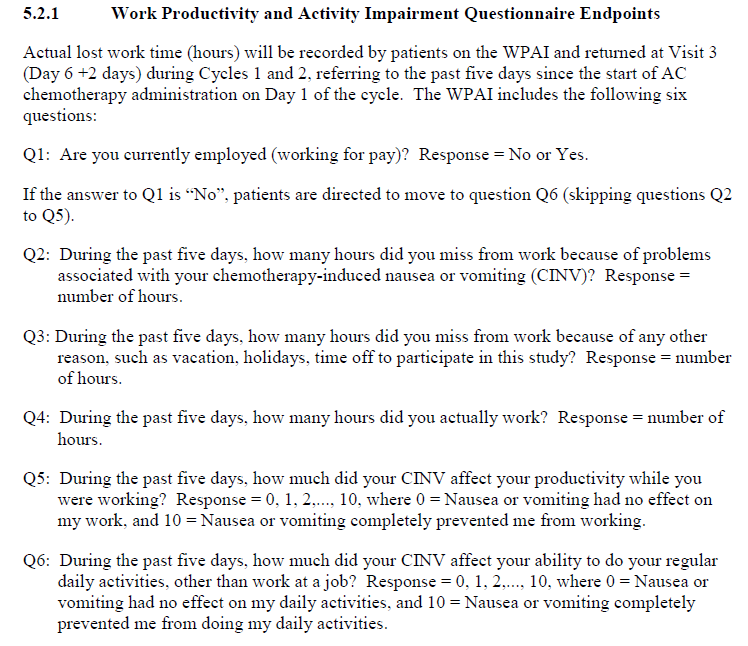

Supplement: Supplementary file 1 — Supplementary file1 (DOCX 124 KB) [file 520_2023_8119_MOESM1_ESM.docx]
